# Sativex in the Management of Multiple Sclerosis-Related Spasticity: Role of the Corticospinal Modulation

**DOI:** 10.1155/2015/656582

**Published:** 2015-01-29

**Authors:** Margherita Russo, Rocco Salvatore Calabrò, Antonino Naro, Edoardo Sessa, Carmela Rifici, Giangaetano D'Aleo, Antonino Leo, Rosaria De Luca, Angelo Quartarone, Placido Bramanti

**Affiliations:** ^1^IRCCS Centro Neurolesi Bonino Pulejo, Contrada Casazza, SS 113, 98124 Messina, Italy; ^2^Department of Neurosciences, University of Messina, Messina, Italy

## Abstract

Sativex is an emergent treatment option for spasticity in patients affected by multiple sclerosis (MS). This oromucosal spray, acting as a partial agonist at cannabinoid receptors, may modulate the balance between excitatory and inhibitory neurotransmitters, leading to muscle relaxation that is in turn responsible for spasticity improvement. Nevertheless, since the clinical assessment may not be sensitive enough to detect spasticity changes, other more objective tools should be tested to better define the real drug effect. The aim of our study was to investigate the role of Sativex in improving spasticity and related symptomatology in MS patients by means of an extensive neurophysiological assessment of sensory-motor circuits. To this end, 30 MS patients underwent a complete clinical and neurophysiological examination, including the following electrophysiological parameters: motor threshold, motor evoked potentials amplitude, intracortical excitability, sensory-motor integration, and H_max_/M_max_ ratio. The same assessment was applied before and after one month of continuous treatment. Our data showed an increase of intracortical inhibition, a significant reduction of spinal excitability, and an improvement in spasticity and associated symptoms. Thus, we can speculate that Sativex could be effective in reducing spasticity by means of a double effect on intracortical and spinal excitability.

## 1. Introduction

Spasticity is frequently experienced by individuals with multiple sclerosis (MS), negatively impacting patient's functional outcomes. Spasticity needs to be carefully assessed and requires long-term management, since it is usually associated with painful spasms, bladder dysfunctions, and pain, increasing the burden of disease [1]. Current therapeutic options are not completely effective in managing such complex symptoms. The medical use of cannabis has generated a lot of interest in the past years, leading to a better understanding of its mechanisms of action. Recently, cannabinoids, such as dronabinol, nabiximols, and nabilone, have been tested for the treatment of spasticity and pain in many neurological diseases. Nabiximols (trade name Sativex) is an oromucosal spray formulation, containing 1 : 1 fixed ratio of delta-9-tetrahydrocannabinol (THC) and cannabidiol (CBD), derived from cloned* Cannabis sativa L.* plant. The main active substance, THC, acts as a partial agonist at human cannabinoid receptors (CB1 and CB2) and may modulate the effects of excitatory (glutamic acid-GLU) and inhibitory (gamma-aminobutyric acid-GABA) neurotransmitters, leading to muscle relaxation with a consequent spasticity improvement [2]. CBD is demonstrated to antagonize some unwanted effects of THC, including intoxication, sedation, tachycardia, anxiety, and other psychoactive symptoms [3]. THC and CBD have a poor bioavailability when orally administrated. However, Sativex is likely to be readily absorbed and to have a good availability, thanks to its sublingual and oromucosal surfaces administration. A previous study showed that Sativex is an effective add-on option for moderate to severe spasticity in MS patients resistant to existing therapies, as demonstrated by its capability to improve spasticity Visual Analog Scale (sVAS) and Ashworth scores [4]. Recent studies showed that Sativex may be effective in improving pain and urinary urgency, since pain VAS (pVAS) and daily number of bladder voids decreased. In addition, some clinical trials evidenced relevant improvements also in quality of life (QoL) [5]. Nevertheless, since Ashworth Scale may not be sensitive enough to detect spasticity changes [6] with consequent discordant effects of cannabinoids on subjective and objective spasticity measures, other more objective tools should be tested to better define the real drug effect. The finding that cannabinoid receptors have predominantly presynaptic rather than postsynaptic effects is consistent with their postulated role in modulating neurotransmitter release [7]. Cannabinoid receptors, in fact, may modulate both excitatory transmission and inhibitory transmission at central synapses and have been heavily implicated in multiple forms of synaptic plasticity, such as long-term potentiation (LTP) and long-term depression (LTD) [8]. Indeed, in a previous study Zachariou and coworkers [8] hypothesized that the activation of cannabinoid receptors by Sativex could modulate the balance between LTP and LTD like plasticity by changing the state of cortical excitability.

The main aim of our study was to better investigate the role of Sativex in improving spasticity in MS patients by means of an extensive clinical-neurophysiological assessment of sensory-motor circuits. The improvement in pain, urinary urgency, and QoL was also evaluated.

## 2. Methods and Materials

### 2.1. Subjects

We selected 47 MS patients attending our Research Institute between January and June 2014, who started treatment with Sativex and matched the following inclusion criteria: age > 18 years, diagnosis of definite MS since at least six months, moderate to severe spasticity in at least two districts of upper and/or lower limbs, absence of clinical or neuroradiological relapses from at least six months prior to study entry, Expanded Disability Status Scale (EDSS) total score >3.5, no history of psychosis, no presence of pace-maker, aneurysms clips, or neurostimulator or brain/subdural electrodes (safety transcranial magnetic stimulation-TMS-procedure). All subjects were taking antispastics, with baclofen being the most common. Only 40% of patients were taking concomitant drugs (mainly analgesics) for other reasons than spasticity. Of the 47 eligible patients, 10 individuals were excluded from the study owing to magnetic and/or electric stimuli intolerance (i.e., 6 patients) or very high resting motor threshold (i.e., 4 patients). Thus, 37 patients were included, at baseline (*T*_0_), in the clinical-electrophysiological study. The experiment was approved by the local Ethics Committee and all subjects provided their written informed consent for the experiments, according to the Declaration of Helsinki.

### 2.2. Experimental Design

The patients underwent a complete clinical-electrophysiological examination at baseline and after one month of continuous treatment, including the assessment of spasticity using the Modified Ashworth Scale (MAS) and the numerical rating scale (NRS), and the evaluation of mobility through the ten-meter walking test (10 WT) and the Ambulation Index (AI). These parameters were considered as primary clinical outcomes. As secondary outcomes, we administered (i) the Expanded Disability Scale (EDDS) for the evaluation of global disability; (ii) pVAS, Penn spasm frequency scale (PSFS), and bladder control scale (BLCS) for spasticity-associated symptoms; and (iii) the Multiple Sclerosis Quality of Life scale (MSQoL-54) to assess patients' QoL. Moreover, we evaluated as primary electrophysiological outcomes the following parameters: short intracortical inhibition (SICI) and facilitation (ICF), and H_max⁡_/M_max⁡_ ratio (H/M) from the abductor pollicis brevis muscle (APB) of the most affected side. Moreover, we also measured the resting (rMT) and active (aMT) motor threshold, the motor evoked potentials (MEPs) amplitude, the cortical silent period (CSP), and the short-latency (SAI) and long-latency (LAI) afferent inhibition from the abductor pollicis brevis muscle (APB) of the most affected side.

### 2.3. TMS Set-Up and Paired-Pulse Measures

MEPs were obtained through magnetic monophasic stimuli delivered through a high-power Magstim200 Stimulator (Magstim, Whitland, Dyfed, UK). The coil was placed tangentially to the scalp with the handle pointing backwards and laterally, at a 45° angle to the sagittal plane, approximately perpendicular to the central sulcus, on the optimal site of the scalp to get the wider MEP amplitude (motor hot-spot), from the APB muscle of the most affected side. The rise time of the magnetic monophasic stimulus was about 100 *μ*s with a to-zero of about 800 *μ*s. The current flowed in handle direction during the rise-time of the magnetic field, thus with a posterior-anterior direction. We preliminarily evaluated the rMT, defined as the smallest stimulus intensity able to evoke a peak-to-peak MEP of 50 *μ*V in rest APB, in at least five out of ten tracks consecutively, and aMT, defined as the minimum stimulator output that produced MEP of 100 *μ*V more in at least 5 of 10 trials with a constant back-ground contraction of 20% of the maximum integrated electromyography [9]. Then, we applied an intensity of stimulation to obtain MEP amplitude of ~0.7 mV. For the CSP we measured the duration of the CSP which is a marker of long-lasting intracortical inhibition (presumably GABA_B_ergic) during slight tonic contraction (~15% of maximum force level) of the target muscle [10, 11]. Audiovisual feedback of ongoing EMG activity was provided to ensure a constant force level. Stimulus intensity was identical to the stimulus intensity used for the TS. EMG traces were rectified but not averaged. The duration of the CSP was measured in each trial and defined as the time from the onset of the MEP to reappearance of sustained EMG activity [12].

Electromyographic activity was recorded through Ag-AgCl surface electrodes applied to APB using a classic muscle belly-tendon montage. Signals were amplified and filtered (from 32 Hz to 1 KHz) via a Digitimer D150 Amplifier (Digitimer Ltd., Welwyn Garden City, Herts, UK) and stored using a sampling frequency of 10 KHz on a personal computer for off-line analysis (Signal Software, Cambridge Electronic Design, Cambridge, UK). SICI and ICF were determined according to the paired-pulse method described by Orth and Rothwell [13]. The intensity of the conditioning stimulus (CS) was set at 80% of aMT. The intensity of the test stimulus (TS) was set to elicit peak-to-peak MEPs amplitude of 0.7 mV. Such intensities were kept constant throughout the experiment. SICI and ICF were assessed at an interstimulus interval (ISI) of 2 and 12 ms, respectively. Mean amplitude of the conditioned MEP was expressed as percentage of the amplitude of the unconditioned MEP and was taken as a measure of corticospinal excitability. We registered in a single trial 15 MEPs, 15 SICIs, and 15 ICFs randomly intermingled. In a separate trial we registered 15 CSPs. All data are given as mean or percentage difference in comparison to baseline values ± standard error (s.e.).

### 2.4. Sensory-Motor Integration

SAI and LAI were explored using the protocol described by Kujirai et al. [14]. An electric CS was given to the correspondent median nerve at the wrist through a Digitimer D-160 stimulator (Digitimer Ltd, Welwyn Garden City, Herts, UK) prior to a magnetic TS given to the contralateral motor area (M1). The median nerve was stimulated through a bipolar-electrode montage at the wrist (cathode-proximal) using a square wave pulse with a pulse-width of 500 *μ*s. The intensity was set just above the threshold for evoking a visible twitch of the thenar muscles (approximately 2.5-times perceptual threshold). The intensity of the TS and the frequency were the same of the SICI protocol. SAI and LAI were probed at ISIs of 25 and 200 ms, respectively. Fifteen stimuli were delivered at each ISI and randomly intermingled with 15 trials in which MEPs were elicited by the TS alone. The mean amplitude of the conditioned MEP was expressed as percentage of the unconditioned MEP mean amplitude. The relative change in MEP amplitude induced by the CS was taken as a measure of the strength of each parameter.

### 2.5. Spinal Excitability Measures

We measured the H-reflex and the H/M ratio evoked in the relaxed flexor radialis carpi (FRC) evoked by the electrical stimulation of the median nerve at elbow (Digitimer D-160 stimulator). Bipolar surface electrodes were applied to nerve's trunk. Skin impedance was kept at less than 10 kΩ. The electrical pulses had a square wave configuration and a pulse width of 1 ms and were applied once every five seconds [15]. The optimal position for stimulating properly the nerve was determined by moving the stimulating electrode around until a visible contraction of the target muscles was observed. Following this procedure, the current was gradually increased until an H-reflex without M-wave was recorded. The largest amplitude response observed without M-wave was designated as the H-max. The stimulus intensity was then further increased in small increments until the maximum M-wave was obtained. The maximum amplitudes of the H-reflex and the M-wave were measured as the difference between the peaks of the positive and negative deflections. The H/M was calculated by dividing the maximum amplitudes of the H-reflex by that of the M-wave.

### 2.6. Statistical Analysis

The Wilcoxon signed-rank tests on the pretreatment/posttreatment (*T*_0_-*T*_30_) scores for the different clinical outcome measures (EDSS, MAS, NRS, 10 WT, AI, pVAS, PSFS, BLCS, and MSQoL-54) were carried out. The alpha level for significance was set at *P* < 0.05. The Bonferroni correction was used for multiple comparisons (*P* < 0.005). The effects of the treatment on rMT, aMT, peak-to-peak MEP amplitude, CSP, paired-pulse intracortical excitability (SICI and ICF), sensory-motor inhibition (SAI and LAI), and H/M ratio were evaluated in separate repeated-measures analyses of variance (ANOVA). For each dependent variable, we computed one-way repeated measures ANOVA with time (two levels: *T*_0_-*T*_30_) as within-subject factors. ISI was considered as an additional factor in the ANOVAs testing changes in paired-pulse intracortical excitability and sensory-motor inhibition. The Greenhouse-Geisser method was used if necessary to correct for nonsphericity. A *P* value <0.05 was considered significant. Post hoc paired-sample *t*-tests were carried out to assess the significance of interactions, applying the Bonferroni correction for multiple comparisons. All data are given as mean or percent ± s.e. We also calculated a Fisher Z-transformation concerning the correlations between the amounts of clinical improvement and of changes obtained by the respective TMS measurements.

## 3. Results

**Figure 1:**
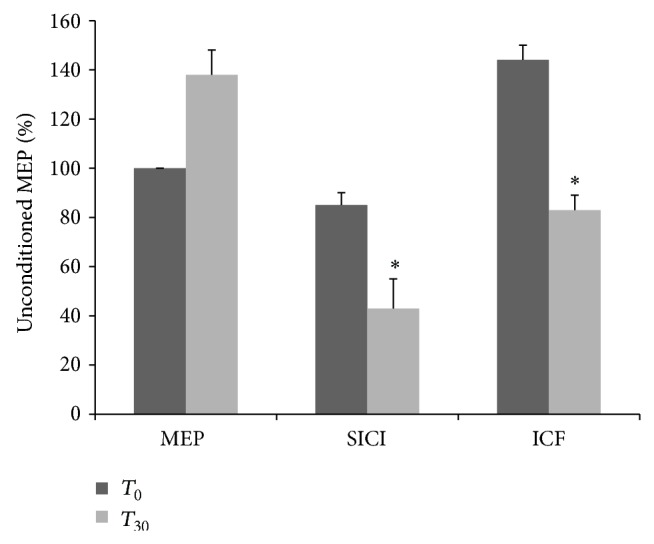
MEP, SICI, ICF, and CSP modifications after one month of Sativex intake.

**Figure 2:**
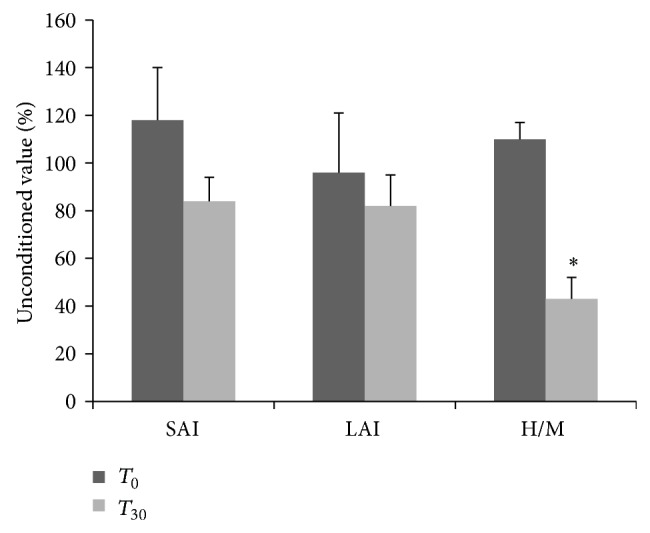
H_max⁡_/M_max⁡_ ratio modification without any changes in SAI and LAI after one month of Sativex intake.

Of the 37 enrolled patients, 7 had to be excluded for drug inefficacy (6 patients) or adverse events (i.e., paranoid ideation). Hence, 30 patients completed the clinical-electrophysiological evaluation and were included in the *T*_0_-*T*_30_ data analysis. After 1 month of nabiximols, the main side effects in the whole sample were dizziness, dry mouth, nausea, and weakness. No significant changes were observed in blood pressure, weight, temperature, hematology, or blood chemistry. Concerning clinical effects after Sativex medication (see Table 1), our cohort of patients showed a significant decrease either in the spasticity subjective scale (*z* = −2.9; *P* = 0.003) or in the objective one (*z* = −2.5; *P* = 0.01). Moreover, we noted a significant improvement in gait parameters, as per 10 wt regarding either the number of patients able to perform the test (*z* = 2.2; *P* = 0.03) or the speed execution (*z* = −2.5, *P* = 0.01), with a concomitant decrease in the AI scores (*z* = −3.2; *P* = 0.002). In addition, we observed a reduction of pVAS (*z* = −2.4; *P* = 0.02), PSFS (*z* = −2.5; *P* = 0.01), and BLCS (*z* = −3.5; *P* < 0.001) scores. No changes were found in rMT (66 versus 69%, *P* = 0.2), aMT (52 versus 55%, *P* = 0.2), and MEP amplitude (*P* = 0.3) (see Figure 1). Our data showed an increased SICI (*F*(1,29) = 15.6; *P* = 0.002) and a reduced ICF strengths (*F*(1,29) = 7.8, *P* = 0.01) (see Figure 1); the CSP duration showed a nonsignificant increase (+20%, *P* > 0.05). Patients showed also a significant reduction of H_max⁡_/M_max⁡_ ratio (*F*(1,29) = 6.3, *P* = 0.05), and no changes instead were found in the others parameters (SAI, *P* = 0.5, and LAI, *P* = 0.5) (see Figure 2). The Fisher test showed significant correlations between SICI and AI (*z* = −2.4, *P* = 0.01), SICI and MAS (*z* = −5.4, *P* < 0.001), and ICF and NRS (*z* = 2.9, *P* = 0.003).

## 4. Discussion

In line with previous studies, we found a significant improvement of spasticity, ambulation, pain, number of daily spasms, and incontinence episodes after a month of nabiximols intake [5, 16, 17]. A new finding of our study is that Sativex can modulate either cortical excitability, as indexed by the increase of SICI and reduction of ICF, or spinal excitability, as showed by the significant (although mild) effect on H/M ratio.

Only few studies have evaluated SICI and ICF changes in individuals affected by MS. Indeed, it has been reported that MS patients, particularly those with a secondary progressive (SPMS) form, had decreased SICI [18, 19]. Interestingly, the SICI reduction seems to be related to EDSS, suggesting that cortical neuronal degeneration or dysfunction may contribute to the development of neurological disability in MS [19]. All these findings imply that intracortical excitability changes may occur in some MS patients, depending on clinical form, degree of disability, and compensatory mechanisms in response to the severity of tissue damage in terms of cortical neuronal loss [20, 21].

To date, no studies have focused on the effect of nabiximols on cortical excitability. To this end, using noninvasive transcranial magnetic and electrical stimulation techniques, it is possible to examine the cortical excitability measures likely involving GABA-_B_ (LICI and CSP) and GABA-_A_ (SICI) receptors, whereas ICF probably reflects the recruitment of excitatory pathways with glutamatergic mediation [22]. It has been shown that high levels of CB1 receptors are associated with inhibitory GABA-interneurons in several brain areas, such as frontal lobes, basal ganglia, cerebellum, hippocampus, hypothalamus, and anterior cingulate cortex [23]. Therefore, we can hypothesize that SICI increases and ICF reduction might be mediated by an effect of Sativex on CB1 receptors. Interestingly, we found significant correlations between several clinical parameters and neurophysiological results, mainly concerning Sativex-induced intracortical excitability modulation (SICI strengthening and ICF weakening).

Human spasticity is related to the reduction of spinal inhibitory mechanisms and, in particular, to reduced Ia afferents presynaptic inhibition [24], and reduced reciprocal inhibition from Ia afferents to antagonist muscles [25, 26]. In addition, abnormal activity of Ib afferents [27] and Renshaw inhibition [28, 29] may also play a role.

One major finding of the present study was the reduction of H/M ratio after Sativex, in line with MAS and NRS scores improvement. The H/M ratio reduction is not surprising since a previous study suggested that the endocannabinoid system may have a prominent role on spinal control, and it may be responsible for the clinical effects on spasticity [30]. On the other hand, it should be acknowledged that, in a different study, H/M rate was not influenced by Sativex, although methodological differences may account for this discrepancy [31].

Nevertheless, we could hypothesize that nabiximols may impact the function of remote spinal circuits, by persistently changing the activity of inhibitory GABAergic corticocortical synapses. In particular, we may speculate that Sativex could modulate the corticospinal projections to local inhibitory interneurons of the spinal cord [24, 32–34], involving the presynaptic control on Ia sensory afferents mediating stretch reflex or the disynaptic reciprocal inhibition [35–37]. To this end, since several reports have shown that repetitive TMS (rTMS) may improve MAS score, probably acting (via corticospinal influence) onto the presynaptic control of Ia sensory afferents mediating stretch reflex [25, 37], one possible scenario would be to use rTMS in association with endocannabinoids to prime and boost up the specific single after-effects on cortical and spinal circuits. The main limitation of our work is that the present study is not placebo-controlled. Therefore, we cannot exclude the possibility of a placebo effect. However, our clinical data are in line with previous findings including a control-group, and it is our opinion that the neurophysiologic changes we found are unlikely to be attributed to a similar placebo effect.

## 5. Conclusion

Our data suggest that Sativex is effective in improving spasticity and related symptomatology probably by modulating cortical excitability through the increase of the inhibitory control over spinal interneurons implicated in spasticity pathophysiology. However, long-term follow-up studies, also including specific electrophysiological protocols, are needed to confirm Sativex efficacy, safety, and drug-related corticospinal excitability changes.

## Figures and Tables

**Table 1:** Clinical effects after one month of Sativex medication. Data are reported as mean ± sd. The asterisk refers to the significant modification at *T*_30_, in comparison to *T*_0_ (^*^ *P* < 0.05, ^**^ *P* < 0.01).

Clinical outcomes		*T*_0_	*T*_30_
Primary	MAS	4 ± 0.7	3 ± 0.9^*^
	AI	7.3 ± 0.5	6 ± 0.6^**^
	NRS	8.3 ± 0.5	5.5 ± 0.4^**^
	10 WT (s)	98 ± 9	69 ± 7^**^
	10 WT (%)	33 ± 8	53 ± 9^**^

Secondary	PFSF	2.8 ± 0.5	2.2 ± 0.3^*^
	MS-QoL	112 ± 8	119 ± 5
	VAS	4.4 ± 0.5	3 ± 0.2^*^
	BLCS	15 ± 2	11 ± 1^**^
	EDSS	6.3 ± 0.2	6.1 ± 0.2

EDDS: Expanded Disability Scale; MAS: Modified Ashworth Scale; NRS: numerical rating scale; 10 WT: ten-meter walking test in seconds of walk-through (s) and percent of patients able to perform the test (%); AI: Ambulation Index; pVAS: visual analogic scale for chronic pain rating; PSFS: Penn spasm frequency scale; BLCS: bladder control scale; MSQoL: Multiple Sclerosis Quality of Life scale.
